# Effects of Combined Transcranial Direct Current Stimulation and Oral Capsaicinoids on Swallowing Function: A Randomized Controlled Trial

**DOI:** 10.1111/joor.70214

**Published:** 2026-05-05

**Authors:** Ivy Cheng, Sonja Suntrup‐Krüger

**Affiliations:** ^1^ Academic Unit of Human Communication, Learning, and Development, Faculty of Education University of Hong Kong Pokfulam Hong Kong; ^2^ Centre for Gastrointestinal Sciences, Faculty of Biology, Medicine and Health University of Manchester Manchester UK; ^3^ Institute for Biomagnetism and Biosignal Analysis University of Münster Münster Germany; ^4^ Department of Neurology University Hospital Münster Münster Germany

**Keywords:** capsaicin, chemical stimulation, pharynx, swallowing, transcranial direct current stimulation

## Abstract

**Background:**

The swallowing biomechanics may be modulated by cortical stimulation through transcranial direct current stimulation (tDCS) or sensory stimulation through oral capsaicinoids. However, the effects of combining these techniques remain relatively unexplored.

**Objective:**

This pilot study investigated the effects of tDCS combined with oral capsaicinoids on swallowing function.

**Methods:**

Twenty‐one healthy individuals (mean age = 25.8 ± 9.9 years; 7 males, 14 females) received two experimental conditions: (1) anodal tDCS and oral capsaicinoids, and (2) sham tDCS and oral capsaicinoids. The experimental conditions were randomized in order and conducted at least 1 week apart. Anodal and sham tDCS were applied over the right pharyngeal motor cortex at 1 mA for 20 min and 30 s respectively. A 15‐min break was given after tDCS, after which participants drank 150 mL of 10 μM capsaicinoid‐containing solution. Swallowing function was assessed using the timed water swallow test (TWST) before, immediately after and 15 min after each experimental condition.

**Results:**

The baseline volume per swallow, duration per swallow and swallowing capacity, as assessed by TWST, were comparable between the two conditions. Combined anodal tDCS and oral capsaicinoids significantly increased the volume per swallow compared with combined sham tDCS and oral capsaicinoids (*p* = 0.003). There were no significant effects of intervention on duration per swallow or swallowing capacity between the two conditions.

**Conclusions:**

We found that combined anodal tDCS and oral capsaicinoids increased the volume per swallow, suggesting potential changes in swallowing biomechanics. The findings imply that cortical stimulation may enhance the effects of peripheral sensory stimulation on the swallowing system.

## Graphical abstract

We investigated the effects of combined transcranial direct current stimulation (tDCS) and oral capsaicinoids on swallowing function in 21 healthy young adults. The combined cortical and peripheral sensory intervention could potentially facilitate swallowing.

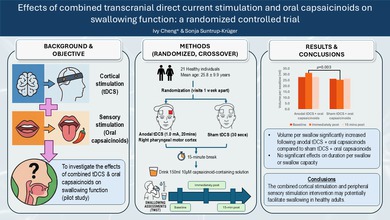

## 1 Introduction

Swallowing is a complex sensorimotor process involving voluntary (oral phase) and involuntary (pharyngeal and esophageal phases) components mediated by the central and peripheral nervous systems [1]. This process is mediated by the central pattern generator in the brainstem and influenced by sensory feedback and input from cortical or subcortical regions throughout the swallowing process [2, 3]. The dynamic interplay between the sensory and motor neural pathways is important for the safety and efficiency of swallowing. Neurological conditions such as stroke can cause damage to neurological structures or pathways involved in swallowing, resulting in dysphagia. Dysphagia can lead to malnutrition, dehydration, aspiration pneumonia, increased risk of secondary infections and mortality [4, 5]. Despite these consequences, the clinical efficacy of dysphagia treatments remains controversial [6].

Neuroplasticity refers to the neural mechanism that enables adaptation to external or internal changes and is important for functional recovery in stroke patients [7, 8]. Hamdy and colleagues showed that unilateral cortical stroke patients with dysphagia who recovered within the first 3 months of stroke onset showed significant increases in the cortical representation and excitability of the pharyngeal (swallowing) motor cortex in the unaffected hemisphere, yet these changes that were not observed in those who did not recover from post‐stroke dysphagia [7, 8]. These findings suggest that neuroplastic reorganization of the swallowing neural network is essential for functional recovery from dysphagia, which lay the foundation for recent treatment approaches that aim to facilitate neuroplastic changes in the swallowing system. Recent studies have showed that transcranial direct current stimulation (tDCS) and transient potential receptor (TRP) agonists may promote neuroplastic changes that are beneficial for neurogenic dysphagia [9, 10]. TDCS modulates the excitability of the motor cortex and induces neuroplasticity by delivering direct electric current onto the brain [11, 12]. When applied on the pharyngeal motor cortex, anodal tDCS upregulates the excitability [13]. When combined with swallowing tasks, anodal tDCS enhanced bilateral cortical activation during swallowing and swallowing function [14].

TRP agonists can activate TRP channels that are widely expressed in the sensory neurons throughout the oropharynx, leading to depolarisation of neurons and triggering of sensory signals in the swallowing system [15, 16, 17, 18]. Studies have shown that capsaicinoid, which is a TRP vanilloid 1 (TRPV1) agonist that can be found in chilli peppers, can enhance swallowing biomechanics, increase salivary substance P level, a neuropeptide known to enhance cough and swallow reflexes, and swallowing function [19]. Importantly, oral capsaicinoids can alter brain activation in regions related to swallowing preparation and stimulus perception, suggesting that sensory input through TRP agonists may have a central effect in the swallowing neural system [20]. Clinically, a systematic review and meta‐analysis showed that TRP agonists have a significant pooled treatment benefit in improving swallowing in patients with post‐stroke dysphagia [10].

Nonetheless, these interventions have limitations. The effects of oral capsaicinoids are dose‐dependent [19, 20]. Single application of oral capsaicinoids may activate the sensory neural pathways peripherally, without modulating the swallowing network at the cortical level [19, 20]. On the other hand, tDCS may induce neuroplasticity changes in the swallowing cortical network, but it does not target specific swallowing pathophysiology. The subtle effects of tDCS in modulating the membrane depolarization threshold instead of directly depolarizing nerve cells [11, 12] may imply that its effects can be more targeted and prominent when combined with peripheral activation of swallowing neural pathways.

Given that both cortical and sensory inputs are critical for the neural control of swallowing, we hypothesize that anodal tDCS combined with oral capsaicinoids may produce greater effects on swallowing function than oral capsaicinoids alone. Therefore, this pilot study aims to explore the effects of combined intervention on swallowing function in young healthy adults.

## 2 Materials and Methods

### 2.1 Ethical Approval

This study was approved by the Human Research Ethics Committee of University of Hong Kong (reference: EA240270). Written informed consent was obtained from all participants. All experimental procedures were conducted in accordance with the Declaration of Helsinki.

### 2.2 Participants

Twenty‐one healthy volunteers (mean age = 25.8 ± 9.9 years; 7 males, 14 females) participated in the study. They had no neurologic, psychiatric, otorhinolaryngological, or swallowing disorders and were not taking any medications affecting the central nervous system. All participants fulfilled the safety criteria for tDCS [21].

### 2.3 Experimental Outline

**FIGURE 1:**
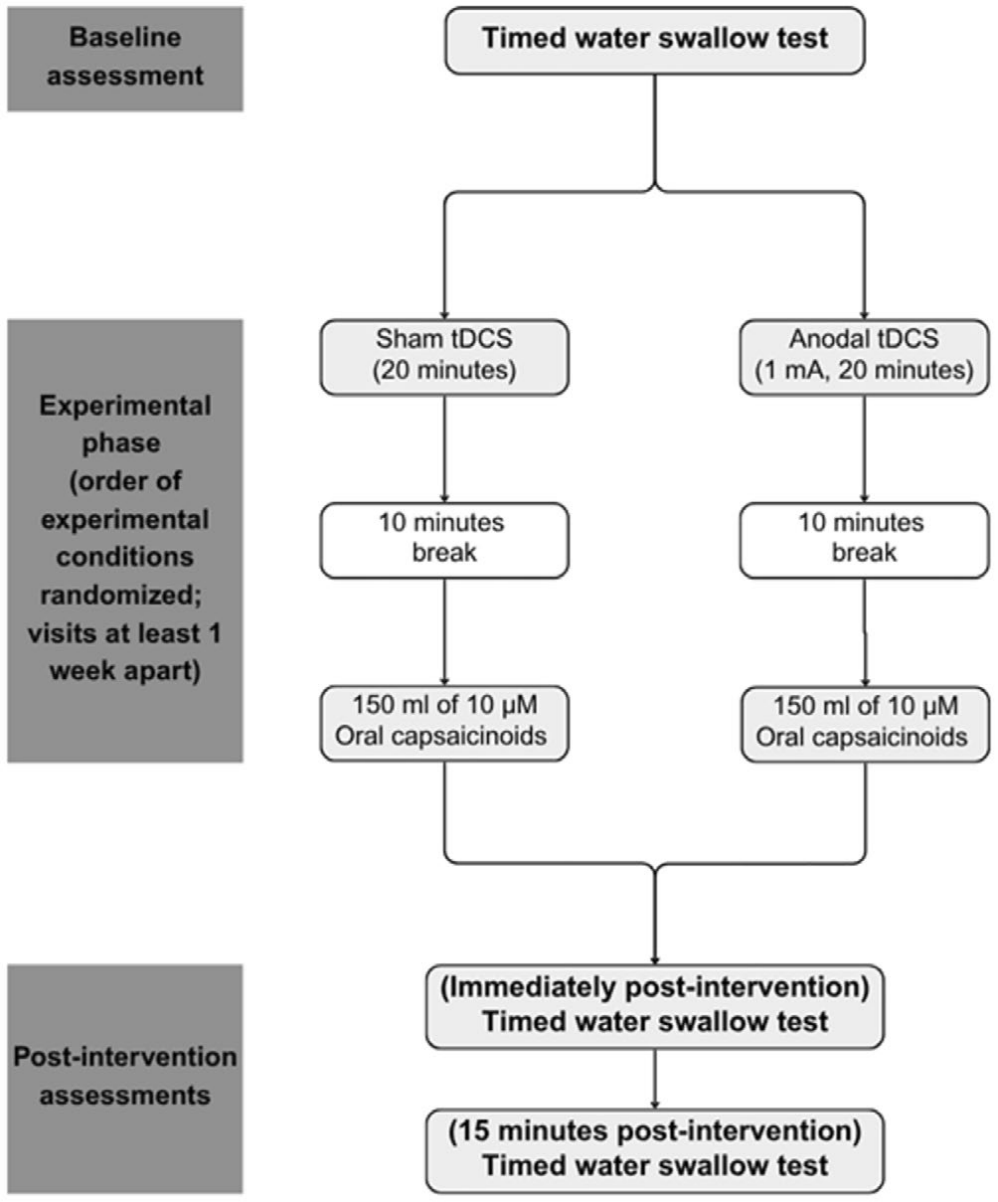
Flow diagram showing experimental outline of the study. TDCS, transcranial direct current stimulation.

This is a randomized controlled study with cross‐over design. Each participant received two experimental conditions: (1) anodal tDCS followed by oral capsaicinoids, and (2) sham tDCS followed by oral capsaicinoids. The conditions were delivered in randomized order on separate visits, with at least 1 week between visits, for each participant. During each visit, participants were seated in an armchair and received anodal or sham tDCS. A rest period of 15 min was given following tDCS. This duration was determined based on previous findings that the maximum effects of anodal tDCS occurred at approximately 15 min [13]. It is hypothesized that the effects of combined anodal tDCS and oral capsaicinoids would be most prominent if oral capsaicinoids were introduced when the effect of tDCS was maximum. Participants then drank 150 mL of capsaicinoid‐containing solution over 5 min to avoid inducing desensitisation effects [19]. According to a previous study, capsaicinoid consumed over 5 min resulted in significant improvements in swallowing biomechanics and performance during challenged swallow task, whereas continuous capsaicinoid consumption led to desensitisation and did not lead to changes in swallowing biomechanics or function [19]. Swallowing function was assessed using timed water swallow test (TWST) [22] before the intervention (baseline), immediately post‐intervention and 15 min post‐intervention. The flow diagram of study procedures is presented in Figure 1.

### 2.4 Experimental Techniques

#### 2.4.1 Transcranial Direct Current Stimulation (tDCS)

A battery‐driven constant current stimulator (NeuroConn GmbH, Ilmenau, Germany) was used to deliver tDCS. A pair of conductive rubber electrodes was placed in two separate sponge pockets pre‐soaked in sodium chloride solution. The anodal electrode had an area of 5 × 7 cm^2^. The centre of the electrode was positioned 3.5 cm lateral and 1 cm anterior to the cortical vertex, corresponding to the pharyngeal motor cortex [7]. The anodal electrode was placed on the right hemisphere, based on previous neuroimaging findings that right‐sided anodal tDCS resulted in more significant enhancement of cortical activation of the swallowing neural network than left‐sided tDCS [14]. The reference electrode had an area of 10 × 10 cm^2^ and was placed over the contralateral supraorbital ridge [23]. Anodal tDCS was delivered at 1 mA for 20 min according to previously established protocol [13]. Sham tDCS was delivered for 30 s only, with the electrodes left in place for a further 20 min. In every session, tDCS current was slowly ramped up to the target intensity over 30 s and ramped down over 30 s at the end of the stimulation. During tDCS, participants were offered room temperature still water to drink to maximize sensory input to cortical swallowing centres and to activate the swallowing network [14]. At the end of the session, they were asked to rate the level of pain and discomfort using a visual analogue scale (VAS) of 1 to 10, with 1 being no discomfort and 10 being intolerably uncomfortable or painful [24]. They were also asked to guess whether they received ‘real’ or ‘sham’ stimulation.

#### 2.4.2 Oral Capsaicinoids

As natural source of capsaicinoids we used commercially available chilli sauce (Tabasco McIlhenny Co, Avery Island, USA). Capsaicinoid‐containing solution was prepared by mixing the chilli sauce with room temperature still water according to the procedures described in a previous study [19]. The concentration of capsaicinoids in the resulting solution was 10 μM. The preparation and consumption of capsaicinoid‐containing solution were thoroughly tested and well‐tolerated in healthy volunteers, as established in a previous study [19].

### 2.5 Timed Water Swallow Test (TWST)

Swallowing function was assessed by TWST [22]. Participants were instructed to drink 150 mL of room temperature still water from a paper cup ‘as quickly as is comfortable’ [22]. An investigator observed from the side while they drank. The duration between the moment when water touched the lips and when the throat came to rest following the final swallow was timed and the number of swallows, as reflected in the number of upward movements of the thyroid cartilage, was counted. The volume per swallow (mL), duration per swallow (s) and swallowing capacity, defined as the time required to drink 150 mL water (mL s^−1^), were calculated for each participant.

### 2.6 Data Analysis

All data were analysed using SPSS Statistics 27.0 (IBM Corporation, USA). Baseline swallowing function based on TWST and VAS for perceived pain and discomfort were compared between anodal and sham tDCS using Wilcoxon signed rank test. Chi‐square test was used to compare the differences in the participants' guesses of ‘real’ versus ‘sham’ between anodal and sham tDCS. Two‐way repeated measures ANOVA was used to evaluate the effects of intervention on swallowing function, including volume per swallow, duration per swallow and swallowing capacity. Greenhouse–Geisser correction was applied when the sphericity assumption was violated based on Mauchly's test. In the case of significant main effects of intervention, the percentage changes from baseline for the parameter were calculated. Post hoc analyses using Wilcoxon signed rank test with Bonferroni adjustments were then performed to compare the differences in the changes from baseline to each post‐intervention time point between the two conditions. Significance was set at *p* < 0.05.

## 3 Results

The experimental procedures were well‐tolerated with no reported adverse effects. Participants reported minimal discomfort (VAS: 1.25 ± 1.66) during tDCS, and they could not distinguish between active and sham tDCS (*p* = 0.367).

### 3.1 Timed Water Swallow Test (TWST)

The baseline volume per swallow, duration per swallow and swallowing capacity were comparable between conditions (Table 1).

**TABLE 1:** Results of timed water swallow test (TWST) performance at baseline and post‐intervention. Data are expressed in mean ± standard deviation.

	Sham tDCS + capsaicinoids	Anodal tDCS + capsaicinoids	Significance
Volume per swallow (mL)			
Baseline	26.21 ± 7.56	27.64 ± 9.39	*p* = 0.306
Immediately post‐intervention	25.35 ± 7.88	31.33 ± 13.03	
15 min post‐intervention	26.49 ± 8.68	27.85 ± 9.54	
Duration per swallow (s)			
Baseline	1.16 ± 0.23	1.22 ± 0.27	*p =* 0.411
Immediately post‐intervention	1.28 ± 0.26	1.31 ± 0.24	
15 min post‐intervention	1.28 ± 0.29	1.17 ± 0.24	
Swallowing capacity (mL s^−1^)			
Baseline	23.79 ± 10.77	22.93 ± 7.15	*p =* 0.821
Immediately post‐intervention	20.37 ± 7.01	24.36 ± 11.05	
15 min post‐intervention	21.33 ± 8.51	24.81 ± 10.88	

Abbreviation: tDCS, transcranial direct current stimulation.

**FIGURE 2:**
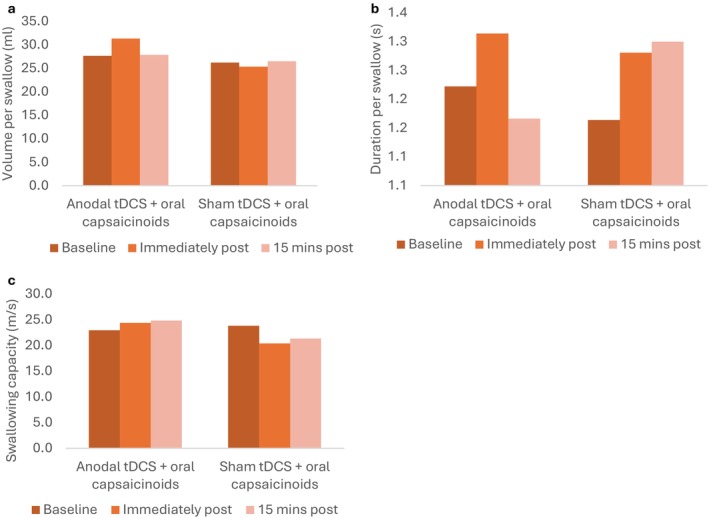
Bar charts showing changes in (a) volume per swallow, (b) duration per swallow, and (c) swallowing capacity over time following the two interventions.

Two‐way repeated measure ANOVA revealed significant effects of intervention (*F*_1,20_ = 5.579, *p* = 0.028, *η*_p_^2^ = 0.218) and interaction (intervention × time) (*F*_2,4_ = 7.711, *p* = 0.001, *η*_p_^2^ = 0.278) on volume per swallow (Figure 2a). This significant interaction indicated that changes in volume per swallow over time differed between the two intervention conditions. Post hoc pairwise comparisons with Bonferroni correction (*α* adjusted to 0.025) showed that the increase in volume per swallow immediately following anodal tDCS and oral capsaicinoids was significantly greater than the change immediately after sham tDCS and oral capsaicinoids (*p* = 0.003). There were no significant differences between conditions in the change from baseline to 15 min post‐intervention (*p* = 0.959).

Regarding duration per swallow, significant effects of time (*F*_2,40_ = 4.295, *p* = 0.020, *η*_p_^2^ = 0.177) and intervention × time interaction (*F*_2,40_ = 4.148, *p* = 0.023, *η*_p_^2^ = 0.172) were observed (Figure 2b). The significant interaction suggested that changes in duration per swallow from baseline to post‐intervention follow‐ups differed between the two conditions. However, post hoc analyses with Bonferroni adjustments (α adjusted to 0.025) revealed no significant differences between conditions in the change from baseline to immediately post‐intervention (*p* = 0.752) or to 15 min post‐intervention (*p* = 0.028).

There were no significant effects of intervention (*F*_1,20_ = 4.152, *p* = 0.055, *η*_p_^2^ = 0.172), time (*F*_2,40_ = 0.612, *p* = 0.547, *η*_p_^2^ = 0.030) or intervention x time interaction (*F*_2,40_ = 2.817, *p* = 0.072, *η*_p_^2^ = 0.123) on swallowing capacity (Figure 2c).

## 4 Discussion

In this study, we investigated the effects of combined anodal tDCS and oral capsaicinoids on swallowing function in healthy adults. Our results showed that the combined intervention led to a short‐term increase in volume per swallow compared to oral capsaicinoids alone. This finding sheds light on the potential benefits in combining non‐invasive brain stimulation with TRP channel agonists, which warrants further discussion.

Our finding supported the hypothesis that combining cortical stimulation and peripheral sensory stimulation enhances the overall effects on swallowing function. This is consistent with previous findings in animal studies that swallowing can be facilitated when cortical stimulation is combined with peripheral electrical stimulation [25]. In rabbits, Sumi showed that swallowing responses induced by stimulation of the anterolateral cortex were facilitated by electrical stimulation of the superior laryngeal nerve [25], suggesting summative effects between the efferent signals descending from the cortex and afferent signals from the peripheral nerves. In humans, peripheral stimulation of the trigeminal or vagus nerves using transcranial magnetic stimulation (TMS) can produce electromyographic responses from pharyngeal and oesophageal muscles, and these responses were facilitated by stimulation of the primary motor cortex [26, 27, 28]. These findings suggest that both descending motor pathways and ascending neural pathways share the same population of motoneurons and interneurons. Anodal tDCS can enhance bilateral activation of the swallowing‐related cortical network [14], excitability of the pharyngeal motor cortex [13], and swallowing behaviour [14]. Importantly, anodal tDCS induces increases in cortical excitability that outlast the stimulation duration, and such excitability changes are thought to be mediated in part by *N*‐methyl‐D‐aspartate (NMDA)‐receptor‐dependent processes and resemble mechanisms of long‐term potentiation, which can contribute to neuroplastic reorganization [11, 12, 29]. On the other hand, a single application of oral capsaicinoids enhances swallowing biomechanics and function, without altering the cortical activation pattern of the swallowing‐related neural network, implying oral capsaicinoid may facilitate swallowing through peripheral activation of sensory neurons in the pharynx [19]. Although the effect of oral capsaicinoids on the swallowing neural network is likely different from electrical stimulation of the pharynx, the activation of sensory neurons may excite the afferent neural pathways, resulting in positive changes in swallowing function. Taken together, the observed changes in swallowing function may result from a summation of excitatory effects between the descending and ascending neural pathways in the swallowing network.

The observed enhancement in volume per swallow following the combined intervention may have positive implications for the older population. Previous studies have reported an age‐related reduction in volume per swallow, as assessed by TWST, in individuals aged 60 and older [22, 30, 31]. Moreover, older adults may show reduced upper esophageal sphincter (UES) opening and relaxation with increasing age [32, 33, 34, 35]. Without instrumental assessment on swallowing biomechanics, we cannot ascertain which parameters, for example pharyngeal constriction pressure or UES opening, contributed to the observed functional change. However, previous studies showed that increased swallowing volume was associated with increased UES diameter [36, 37]. Moreover, oral capsaicinoids could enhance pharyngeal swallow vigour and prolong UES relaxation time [19]. It is plausible that anodal tDCS enhanced the effects of oral capsaicinoids, leading to an increase in volume per swallow that was associated with an increase in UES opening. However, given that our findings were based on a functional test (TWST), this interpretation remains speculative. These results should be regarded as exploratory and provide the basis for future studies that incorporate instrumental assessments such as high‐resolution pharyngeal manometry (HRM) or flexible endoscopic evaluation of swallowing (FEES) to investigate the underlying biomechanical changes that drive the observed functional outcomes.

Of note, while the observed outcome of increase in volume per swallow may be interpreted as optimized swallowing function in young healthy volunteers, it may not be beneficial in older individuals. Presbyphagia, which limits one's capacity to manipulate bolus safely and efficiently, may be present in older adults. Consequently, an increase in volume per swallow may increase the difficulty in bolus control, leading to an increased risk of aspiration. A similar impact may occur in patients with dysphagia who have impaired bolus control. Therefore, further studies should be performed in the older population and patients with dysphagia to determine the clinical benefits and potential risks of the combined intervention on swallowing safety and efficiency.

Finally, the observed changes following the combined intervention were transient, lasting less than 15 min. This short duration may reflect a limited effect on a healthy functional swallowing system. However, the transience of effect warrants further investigation into whether repeated dosing is required to achieve a sustained clinical benefit.

Our study has several limitations. First, our study lacks a full factorial design as we did not include oral capsaicinoid‐only or tDCS‐only conditions. Since oral capsaicinoids were administered in both conditions, it cannot be ascertained whether the observed effects on volume per swallow reflected an interaction between cortical and peripheral inputs, an additive enhancement of oral capsaicinoids by tDCS, or a tDCS effect that occurs only in the presence of oral capsaicinoids. Although our interpretation refers to findings from studies of oral capsaicinoids or tDCS alone, the true individual and combined effects cannot be delineated without head‐to‐head comparisons within a 2 × 2 factorial design. Second, as a pilot study, we focused on a relatively young cohort, which was appropriate for exploring the potential effects of the combined intervention on swallowing. Given that swallowing neurophysiology and neuroplastic mechanisms change with age [38, 39, 40, 41], the generalizability of our findings to older adults and dysphagic patients may be limited. Moreover, objective physiological or neurological measurements were not used in the study. Nonetheless, the participants' swallowing function was effectively assessed by using a functional test in a naturalistic setting. Finally, in this pilot study, we measured changes in swallowing function at only two time points: immediately and 15 min post‐intervention. This limited timeline may not fully capture the dynamic effects of the intervention. Future studies should include additional time points to better evaluate transient changes over time.

## 5 Conclusions

In summary, this preliminary study showed that combined anodal tDCS and oral capsaicinoids may potentially facilitate swallowing in healthy adults. The results suggested that cortical stimulation combined with peripheral sensory stimulation led to a short‐term increase in volume per swallow as assessed by functional swallowing test. Future studies may explore the physiological and neurological effects of the combined intervention and its therapeutic potential in the older population and in patients with neurogenic dysphagia.

## Author Contributions

**Ivy Cheng:** conceptualization, funding acquisition, investigation, writing – original draft, methodology, validation, visualization, writing – review and editing, formal analysis, data curation, resources, project administration. **Sonja Suntrup‐Krüger:** conceptualization, funding acquisition, methodology, validation, formal analysis, writing – review and editing, supervision.

## Funding

This study was supported by the Faculty Research Fund from the Faculty of Education, University of Hong Kong, awarded to the first author. The senior author is supported with an endowed professorship from the Else Kröner‐Fresenius‐Stiftung.

## Conflicts of Interest

The authors declare no conflicts of interest.

## Data Availability

The data that support the findings of this study are available from the corresponding author upon reasonable request.
